# Modulation of the neuronal response in human primary visual cortex by re-entrant projections during retinal input processing as manifest in the visual evoked potential

**DOI:** 10.1016/j.heliyon.2024.e30752

**Published:** 2024-05-09

**Authors:** Valentine L. Marcar, Martin Wolf

**Affiliations:** a University Hospital Zürich, Biomedical Optics Research Laboratory (BORL), Frauenklinikstrasse 10, CH-8091, Zürich, Switzerland; b University Hospital Zürich, Comprehensive Cancer Center Zürich (CCCZ), Rämistrasse 100, CH-8091, Zürich, Switzerland

**Keywords:** Temporal - and spatial luminance contrast, Local and global stimulus properties, Afferent and efferent input, Feature extraction, Object closure

## Abstract

Initial deflections in the visual evoked potential (VEP) reflect the neuronal process of extracting features from the retinal input; a process not modulated by re-entrant projections. Later deflections in the VEP reflect the neuronal process of combining features into an object, a process referred to as ‘object closure’ and modulated by re-entrant projections. Our earlier work indicated that the VEP reflects independent neuronal responses processing temporal – and spatial luminance contrast and that these responses arise from an interaction between forward and re-entrant input. In this earlier work, changing the temporal luminance contrast property of a stimulus altered its spatial luminance contrast property. We recorded the VEP in 12 volunteers viewing image pairs of a windmill, regular dartboard or an RMS dartboard rotated by either Π/4, Π/2, 3Π/4 or Π radians with respect to each other. The windmill and regular dartboard had identical white to black ratio, while the two dartboards identical contrast edges per unit area. Rotation varied temporal luminance contrast of a stimulus without affecting its spatial luminance contrast. N75, P100, N135 and P240 amplitude and latency were compared and a source localisation and temporal frequency analysis performed. P100 amplitude signals a neuronal response processing temporal luminance contrast that is modulated by re-entrant projections with fast axonal conduction velocities. N135 and P240 signal the neuronal response processing spatial luminance contrast and is modulated by re-entrant projections with slow axonal conduction velocities. The dorsal stream is interconnected by fast axonal conduction velocities, the ventral stream by slow axonal conduction velocities.

## 1 Introduction

“In the middle of difficulty lies opportunity”.

Albert Einstein (1879–1955)

The introduction of non-invasive means of visualising brain activity has greatly aided our understanding of the neuronal processes of cognitive function in humans. Functional magnetic resonance imaging (fMRI), using the blood oxygenation level dependent (BOLD-) signal, has revealed the macro-neuronal network involved in cognition and perception with high spatial resolution. Arising from a drop in local deoxyhaemoglobin level following a local increase in blood flow that peaks after 6s [1], the BOLD signal is unsuitable for capturing a fast changes in neuronal response. Changes in neuronal response at the millisecond level are captured using electroencephalography (EEG) or magnetoencephalography (MEG). The former by recording the electric potential at the scalp the latter through changes in the local magnetic field. Ease of use and low cost has made EEG the preferred option to study neuronal processing in health individuals as well the influence of viral infection [2], autoimmune reaction [[3], [4], [5]], genetic disease [6,7], ageing [8], neuropathy [9,10] and atypical cognitive development [11] on neuronal processing. Although EEG offers a poorer spatial resolution than fMRI, by sampling the electric potential from standardised locations across the scalp [12] it is possible to identify the Brodmann area of an electric signal with a reasonable degree of accuracy [13].

Isolating the electric potential generated by the neuronal response accompanying the processing of a specific pattern or task involves averaging the electric potentials from repeated occurrences of that stimulus or task, resulting in the evoked potential (EP). Averaging enhances the electric potentials locked to a stimulus or task and attenuates those that are not [12,14]. The EP is viewed as a reflection of the strength and direction of flow of the extracellular, ionic current flowing between apical dendrites and soma of pyramidal cells [15]. This current is linked to the local field potential arising from the summed influence of all excitatory and inhibitory post synaptic potentials acting at the apical dendrites [16].

Deflections in the EP are referred to as ‘components’ and are characterised by the time the deflection peaks, i.e. its latency and its amplitude. An EP reflects the change in activity of a large number of neurons rather than that of a select population [17], and so represents a mass-action response. While the neuro-physiological mechanisms of an EP are well understood, the relationship between stimulus property or task difficulty, the neuronal response they elicit and the EP is less clear. Such an understanding is key to inferring a change in neuronal response from a change in EP. With much known about the anatomical and functional organisation of the primate visual system it is a frequent site for investigating the relationship between stimulus property, neuronal response and the visual evoked potential recorded over the occipital pole (VEP) [12]. VEP components with a latency of 100 ms or less are regarded as exogenous, i.e. a neuronal response driven by basic neuronal mechanisms and not subject to modulation by re-entrant projections. Components with a latency beyond 100 ms are considered endogenous, i.e. driven by internal factors and cognitive mechanisms [18]. This neuronal response is considered subject to modulation by re-entrant projections. In the case of the neuronal response in V1 modulation by re-entrant projections originating in areas of extra-striate cortex [[19], [20], [21], [22], [23]]. Primate visual cortex is divided into striate cortex (V1) and a collection of extra-striate areas. The latter consists of numerous cortical areas interconnect in manner yielding a dorsal- and ventral processing stream processing the retinal input in parallel [24]. The signal from retinal ganglion cells, relayed via the dorsal lateral geniculate nucleus of the thalamus, activates neurons in V1 within 50 ms. This thalamic activity is modulated within 15 ms by re-entrant projections from V2 [25,26]. This implies that any neuronal response captured by the VEP during retinal input processing in the visual system is the result of recursive interaction between forward and re-entrant signals [27]. It is therefore not possible to attribute a neuronal response to thalamic activation alone, as it always has been modulated by re-entrant projections. Studies of retinal input processing in human visual cortex using electrophysiology [[28], [29], [30], [31]] and transcranial magnetic stimulation (TMS) in human volunteers [32], as well as neurophysiological recording in the monkey [33] described two distinct activation periods in V1. Psychophysical work identified a transient and a sustained response during processing within the human visual system [34]. Theoretical models of visual object perception envisage an initial stage extracting features, such as edges and corners, in the retinal input [35,36], followed by a subsequent stage where identified features are combined into an object; a process referred to as ‘object closure‘ or ‘perceptual closure’ [37,38]. Extracting features matches the selectivity of V1 neurons [39]. Combining these features into an object involves linking these features, a process where the response of V1 neurons by re-entrant projections originating in extra-striate cortex [[40], [41], [42]]. Extraction of features and ‘object closure’ both involve a mechanism selective to spatial luminance contrast, i.e. a change in luminance between two locations in space. Our earlier work noted a linear relationship between the between N75 and P100 amplitude. This led us to conclude that their amplitude reflected the size of the neuronal population active during temporal luminance contrast (δI/δt) processing. The same work noted that N135 and P240 amplitude reflected the size of the neuronal population active during the spatial luminance contrast (δI/δs) processing [31]. Later studies demonstrated that the electric potential arising from these two mechanisms are of opposite polarity and interfered destructively, such that increasing the neuronal response during spatial luminance contrast processing attenuated P100 amplitude [43,44]. The VEP from the same pattern viewed as a pattern reversing and on-off stimulus found N75 and P100 amplitude to be the same but N135 and P240 amplitude to be larger for the latter. This points to N75 and P100 signalling a phasic neuronal response and N135 and P240 a tonic neuronal response [43,45,46]. It also demonstrated that the nature of the neuronal response determines its ability to become manifest in the VEP. In this preceding work we varied made use of the retinotopic organisation of V1 to vary the size of the neuronal population active during temporal luminance contrast processing by changing the relative stimulus area undergoing a change from black to white. However, this invariably altered the form of the elements comprising each pattern and with it the re-entrant signal modulating the neuronal response in V1. We therefore aimed to investigate the effect of re-entrant projections on the neuronal response during temporal luminance contrast processing by including a condition where the re-entrant signal was kept constant. We compared the VEP from volunteers to three patterns, a windmill, a regular dartboard and the RMS version of the dartboard. These patterns were designed such that the windmill and regular dartboard had the same temporal luminance contrast and two dartboards the same spatial luminance contrast [46]. In order to vary the size of the neuronal population processing temporal luminance contrast but keeping the re-entrant signal constant, we presented them using the spatial-phase paradigm of Ratliff and Zemon [47]. In this paradigm image pairs of the same pattern were viewed rotated through a specific angle, i.e phase angle relative to each other. Increasing phase angle increased the relative area of white changing from black to white and so the number of neurons active during temporal luminance contrast processing. For a given pattern the number of neurons active during spatial luminance contrast processing was constant. We predicted that if the neuronal response signalled by P100 that has spatial luminance contrast characteristics, its amplitude would remain constant across phase angle. This indicates that its neuronal response is modulated by a signal that arises after object closure. P100 amplitude is predicted to increase linearly with spatial phase if modulation of the neural response by re-entrant projections involves a signal that arises before object closure.

## 2 Materials and methods

Hypothesis1 Null Hypothesis (H0): P100 amplitude does not change with phase angle.

Alternative Hypothesis (H1): P100 amplitude increases with rotation phase angle.

Participants: Twelve healthy, adult volunteers (8 ♂), aged between 23 and 36 years (mean 27 years; σ = 4.2 years) participated in the study and represent a convenience sample. All had had normal visual acuity, an education at tertiary level and stated that they were not taking any prescription medication for any physiological or suffered from a psychiatric health issue. Data published in Ref. was collected concurrently with the data reported here. The study protocol was approved by the local ethics committee (KEK-ZH E−50/2002) and conformed to the requirements of the 2008 revision of the World Medical Association Helsinki Declaration. All participants provided their written, informed consent.

### 2.1 Apparatus

Monitor: The stimuli were presented on a 22″ monitor (Dell ST2210B; Round Rock, TX, USA) set to a spatial resolution mode of 1650 by 1050 pixel and refresh rate of 60 Hz. The monitor's brightness was set to 80 %, the contrast to 90 %.

Stimuli: A detailed description of the properties of the patterns used is given in Ref. [46]. Only brief description of the main properties is provided here. The ratio of white to black area of windmill and regular dartboard was 50:50 in both. Viewed in terms of spatial frequency properties the two patterns had the same power in the f(0) function. Given the retinotopic organisation of V1, the two pattern result in the same ratio of cortical areas within which neurons are active and inactive. The stimulus area undergoing a change from black to white corresponds to the temporal luminance contrast property of these patterns, so the size of the neuronal population activated by their temporal luminance contrast was the same. The area undergoing a change from black to white was much smaller in RMS dartboard. Consequently, its temporal luminance contrast property was much smaller and with it the size of the neuronal population active during processing. The regular - and RMS dartboard had the same number of contrast edges or luminance contrast per unit area [48]. The two patterns had identical spatial luminance contrast properties and the same summed power in the spatial frequencies to which humans exhibit the highest contrast sensitivity i.e. 3-7 cycles/degree. The size of the neuronal population activated by their spatial luminance contrast was the same. This contrasts with the spatial luminance contrast of the windmill, which was smaller and hence activated a smaller neuronal population. Element size increased with eccentricity in order to compensate for the increase in receptive field size. The luminance of the white elements was 252 cd/m^2^, that of the black elements a luminance of 3.4 cd/m^2^ (Minolta: LS 110; Osaka, Japan), corresponding to a Michelson contrast of 97.3 in all images. At the viewing distance of 0.83 m, the patterns occupied the central 10° of the visual field. All 12 participants viewed all 12 displays binocularly with their head positioned on a chin and forehead rest (Richmond Products Inc., Albuquerque, NM, USA).

**Fig. 1:**
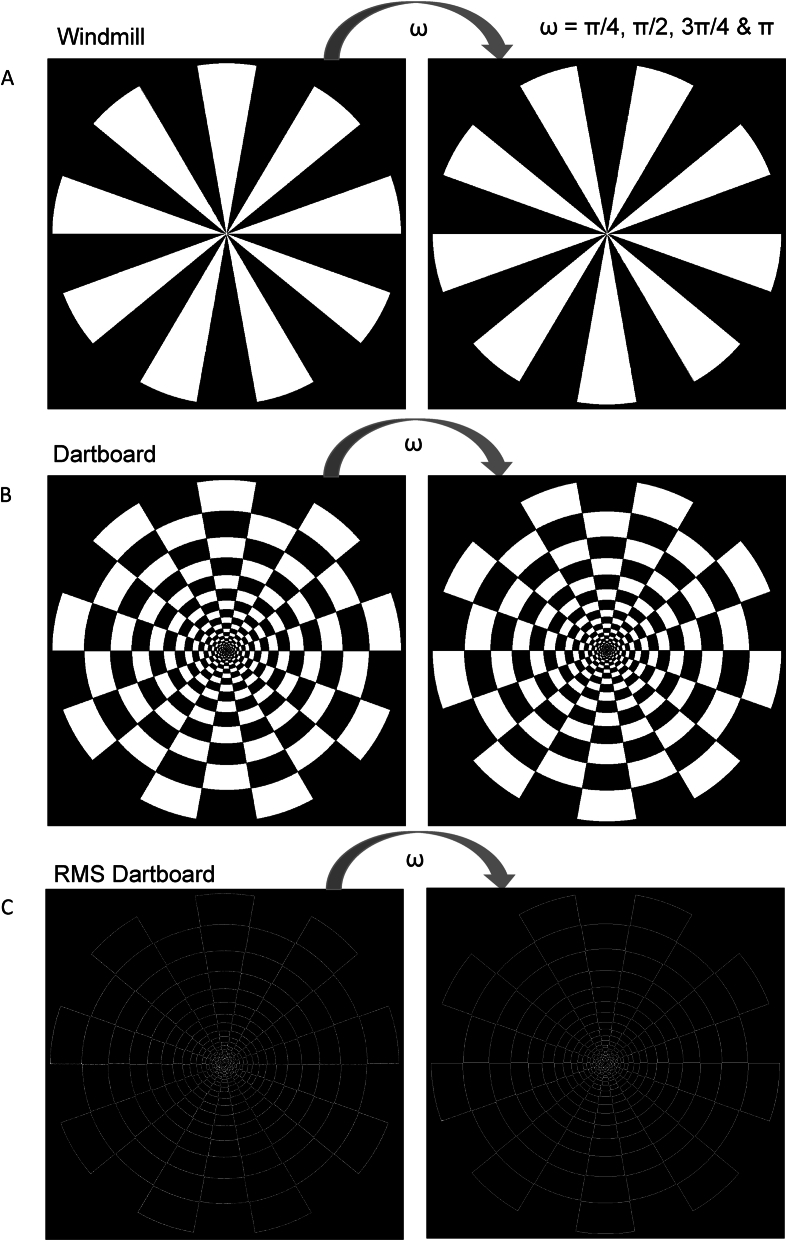
Pannel A shows the windmill pattern, panel B shows the regular dartboard pattern and panel C shows the RMS dartboard pattern used in our study. The two images in each panel illustrate how image pairs were used to generate our pattern reversing stimuli by shifting their phase with respect to the other. The image pairs depicted have a phase shift of Π. See text for an extensive description of the stimuli.

Each pattern reversing displays consisted of a pair of images presented in alternation where the pattern in the two images were rotated by Π/4, Π/2, 3Π/4 or Π radians with respect to the other. Increasing the rotation angle increased the phase angle between them. This led to the relative stimulus area changing from black to white increasing with increasing phase angle. The area of a disc of radius equal to that of the dartboard or windmill represented 100 %. At a phase angle of Π/4 radians the stimulus area changing from black to white amounted to 12.5 %. At Π/2 radians it amounted to 25 %, at 3Π/4 radians to 37.5 % and at Π radians to 50 %. During a pattern reversing displays image pairs were exchanged every 500 ms for 60s; resulting in 120 reversals. Fig. 1 depicts the three types of images used.

To avoid an order effect, stimulus sequence was randomised using the Latin square method between participants.

Electrophysiology: EEG measurements were performed in the laboratory of the Biomedical Optics Research Laboratory at the University Hospital Zürich and followed the guidelines of the International Society for Clinical Electrophysiology of Vision (ISCEV) of 2009 [12]. Electric potentials were recorded using 32 active electrodes distributed across the scalp following the International 10/10 system [49,50]. To this end, electrodes were placed in an electrode cap (ActiCap 32, MES, Munich, Germany). Electrodes were located at the following sites: Fp1/2, F7/8, F3/4, FC5/6, Fz, FC1/2, T7/8, C3/4, Cz, TP9/10, CP5/6, CP1/2, P7/8, P3/4, Pz, PO9/10, O1/2 & Oz. In addition, electrodes AFz and FCz served as ‘GROUND’ and ‘REFERENCE’ respectively. Electrode impedance was kept at or below 5 kΩ and sampling rate was set to 500 Hz. Electric potentials at each electrode were stored on the HDD drive of a PC using commercial software (Brain Vision Recorder, Brain Products, Munich, Germany) running under Windows 7 (Microsoft, Redmond, WA, USA). During a recording session the monitor provided the only source of illumination.

Data processing: Offline processing of the EEG data was performed using ‘Vision Analyser’ (Ver. 2.1, Brain Products, Munich, Germany). The EEG data was bandpass filtered so that oscillations below 0.1 Hz and above 40 Hz and with a slope less than 24dB/oct and above 48dB/oct were removed. For each time point, we averaged the signal from all electrodes and re-referenced the signal of each electrode with respect to this average. Artefacts due to blinking were identified using the independent component analysis (ICA) module of Vision Analyzer 2.1 and removed. Lastly, any remaining artefacts were identified by visually and marked manually.

The visual evoked potential (VEP): The signal from electrode Oz was selected, as it is closely associated with neuronal activity in V1 [12,51,52]. To obtain the VEP for a pattern at a specific phase angle, data segments of 500 ms from the pattern at the phase angle were averaged, starting at an image exchange. The amplitude and latency of the four VEP component N75, P100, N135 and P240 reported by Marcar and Jäncke were noted [31]. N75 amplitude was the minimum electric potential between 50 ms and 80 ms, that of P100 the maximum between 70 ms and 120 ms, that of N135 as the minimum between 100 ms and 180 ms and that of P240 the maximum between 180 ms and 300 ms. The time point at which the maximum or minimum occurred served as their latency.

Statistical analysis: VEPs from each participant were obtained under several related conditions, so that they effectively served as their own reference between conditions. We assessed differences in VEP amplitude and latency use the repeated measures ANOVA of JASP (JASP Team 2020, Ver. 0.14, [Computer Software], University of Amsterdam, Netherlands). The factors were “PATTERN” (WM, DB & RMS DB) and “PHASE ANGLE” (П/4, П/2, 3П/4 & П). In order to reduce the risk of a Type I error, we set the threshold for a difference between conditions as p ≤ 0.01. Partial ETA^2^ (η_p_^2^) served as our measure of effect size.

Neuronal source localisation: The site of highest current source density (CSD) was identified for each VEP component using the approach implemented in sLORETA-KEY. This method uses electric potentials between adjacent electrodes to calculate the current source density between them and does not require any a priori assumptions [53]. It has a spatial accuracy of 7 mm [54] sufficient to identify the Brodmann area of a VEP component. A detailed description of the method upon which LORETA is based is given in Ref. [55], so that only an outline will be provided here. First, a 3-dimensional distribution of current density is calculated for each time point based on the electric potentials of all 32 electrodes using a linear solution constrained by the requirement that the map is the smoothest solutions available. The current density maps are then projected onto a normalised brain with a volume of 2394 voxels using a three shell spherical model. To visualise the current density distribution for each of our conditions we averaged the CSD maps across all 12 participants. The topographic analysis of variance (TANOVA) module of LORETA enables an assessment of global difference in current density maps. This module performs a voxel-by voxel *t*-test of the current density maps from different conditions. This approach involves a non-parametric estimation of the probability distribution of the maximum of the t-statistic against a set of random distribution. Correction for multiple-testing employs the Bonferroni method. The non-parametric nature of this approach renders it free of the constraints imposed by of normal data distribution. We used TANOVA to compared the CSD maps from the windmill and RMS dartboard pattern to identify differences in current density associated with temporal - and spatial luminance contrast processing, as the temporal – and spatial luminance contrast properties in these two pattern was largest. This comparison was performed using the paired group module in the LORETA. CSD values were not normalised but smoothed across time using a factor 0.3. All t-values were based on a comparison against 20′000 randomisations. We accepted t-values with a p-value ≤0.05 as indicating the presence of a difference.

Temporal frequency analysis: Work by Frund and colleagues linked high spatial frequency processing in the human visual system to a prominence in low temporal frequencies in the VEP and vice versa [56]. In order to examine the presence of such a link in our data, we determined the time/frequency composition of the VEP across time, using the ‘Wavelet’ module in Vision Analyzer. The ‘c’ parameter of the ‘Morlet’ filter was 3.8, minimum frequency was 1 Hz, maximum frequency 40 Hz and step size 1 Hz. The time/frequency spectrum from the 12 participants were then averaged within each condition and the mean time/frequency spectrum of each VEP displayed in the form of a Winger plot and saved.

Lastly, we extracted peak power present in the alpha (8–13 Hz) and beta (14–30 Hz) band and compared it between conditions using a repeated measures ANOVA with the factors PATTERN and PHASE ANGLE.

## 3 Results

Comparing individual VEP to the three patterns.

**Fig. 2:**
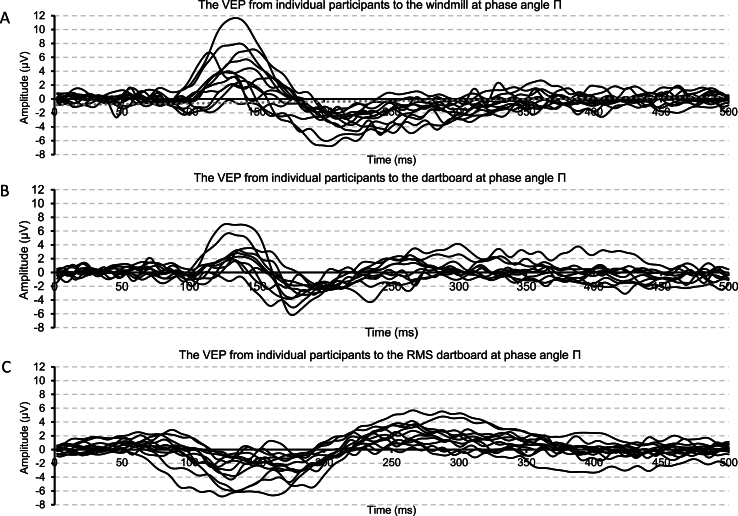
Panel A depicts individual VEP to the windmill pattern rotated through Π radians. Panel B depicts individual VEP to the regular dartboard rotated through Π radians. Panel C depicts individual VEP to the RMS dartboard rotated through Π radians.

Panels A–C of Fig. 2 show the individual VEP at electrode Oz to the three patterns when viewed in a pattern reversing display and a phase angle of Π radians.

### 3.1 Comparing the grand, mean VEP to the three patterns

**Fig. 3:**
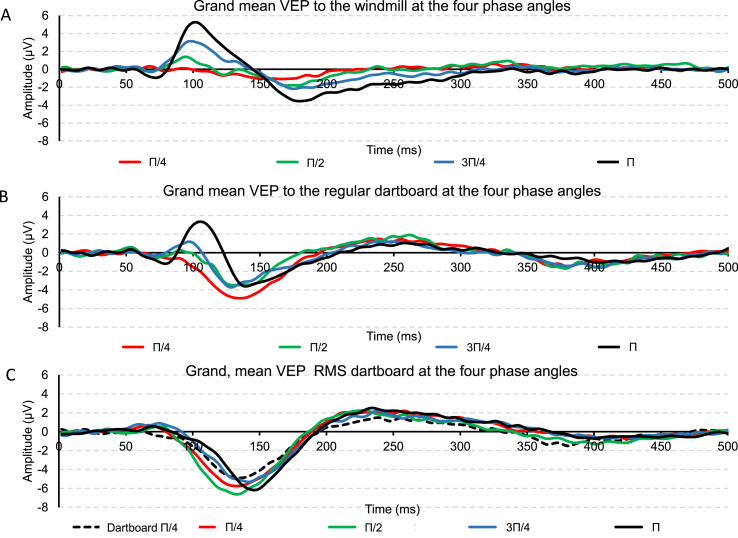
Panel A depicts the grand, mean VEP when the windmill was rotated through the four different angles. Panel B depicts the grand, mean VEP when the regular dartboard was rotated through the four angles. Panel C depicts the grand, mean VEP to the RMS dartboard was rotated through the four angles. This panel also contains the grand mean VEP obtained to the regular dartboard when rotated through Π/4 radians.

Panels A–C of Fig. 3 show the grand mean VEP at Oz to the three patterns at each phase angle.

### 3.2 Comparing VEP component amplitude

**Fig. 4:**
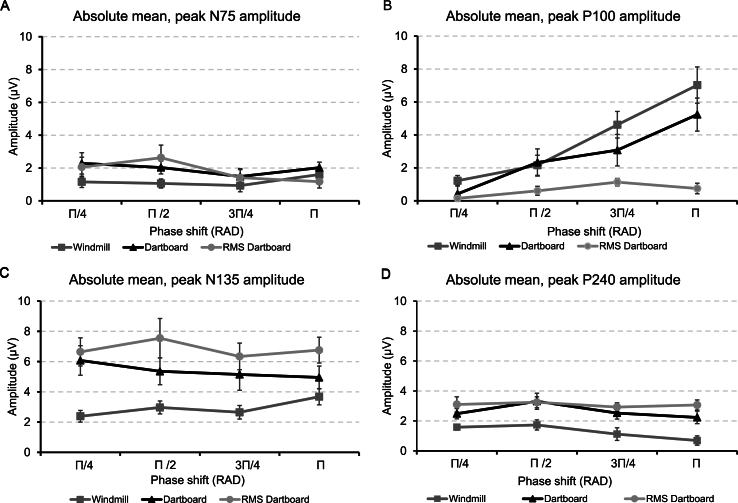
Panel A shows the mean, peak amplitudes of the N75 VEP component to the three patterns when rotated through the four angles. Panel B shows the mean, peak amplitudes of the P100 VEP component to the three patterns when rotated through the four angles. Panel C shows the mean, peak amplitudes of the N135 VEP component to the three patterns when rotated through the four angles. Panel D shows the mean, peak amplitudes of the P240 VEP component to the three patterns when rotated through the four angles. The error bars indicate the standard error of the mean.

Panels A–D of Fig. 4 show the grand mean amplitude of the four VEP components to the three patterns at each phase angle.

Table 1 contains the results of the comparison of the individual VEP components (see Table 1).

**Table 1:** The table holds the results from the repeated measures ANOVA of individual VEP component amplitude to the three pattern and four phase angles. Violation of sphericity was corrected by adjusting the degrees of freedom using the method of Huynh-Feldt.

	F	df	df Residuals	p	η^2^
**N75**					
Pattern	1.171	2.000	33.000	0.323	0.040
Phase angle	2.167	3.352	85.491	0.097	0.022
Pattern*Phase angle	1.746	5.181	85.491	0.130	0.035
**P100**					
Pattern	8.849	2.000	33.000	<0.001	0.176
Phase angle	32.557	2.044	67.439	<0.001	0.206
Pattern*Phase angle	6.561	4.087	67.439	<0.001	0.083
**N135**					
Pattern	7.059	2.000	33.000	0.003	0.250
Phase angle	0.971	2.630	80.443	0.397	0.004
Pattern*Phase angle	1.750	4.857	80.443	0.134	0.015
**P240**					
Pattern	8.678	2.000	33.000	<0.001	0.254
Phase angle	5.539	2.770	91.395	0.002	0.035
Pattern*Phase angle	1.129	5.539	91.395	0.352	0.015

N75 amplitude did not change between the three pattern nor across phase angle. No two-way interaction between the factors PATTERN and PHASE ANGLE was present. P100 amplitude to the windmill and regular dartboard exceeded that to the RMS dartboard. It increased with increasing phase angle. A two-way interaction between the factors PATTERN and PHASE ANGLE was present. N135 amplitude to the two dartboards exceeded that to the windmill but did not change across phase angle. No two-way interaction between the factors PATTERN and PHASE ANGLE was present. P240 amplitude to the two dartboards also exceeded that to the windmill but decreased with increasing phase angle. No two-way interaction between the factors PATTERN and PHASE ANGLE was present.

Table 1a contains the results of our post-hoc comparison of component amplitude to the different conditions.

**Table 1a:** Result of post hoc analysis of VEP component amplitude at the four phase angles and 3 patterns. All p-values adjusted for comparing a family of 6 phase angles or 3 patterns using the method Holm-Bonferroni. Phase angle results are averaged over PATTERN and pattern results are averaged over PHASE ANGLE.

Post Hoc Test				
N75	Mean Δ	SE	t-value	p_holm_
Π/4 vs Π/2	0.071	0.327	0.218	1.000
Π/4 vs 3Π/4	−0.558	0.327	−1.704	0.489
Π/4 vs Π	−0.234	0.327	−0.714	1.000
Π/2 vs 3Π/4	−0.629	0.327	−1.922	0.380
Π/2 vs Π	−0.305	0.327	0.932	1.000
3Π/4 vs Π	0.324	0.327	0.990	1.000
Dartboard vs RMS Dartboard	−0.139	0.456	−0.305	0.763
Dartboard vs Windmill	0.769	0.456	1.687	0.317
RMS Dartboard vs Windmill	0.630	0.456	1.382	0.362

**Post Hoc Test**				
**P100**	**Mean Δ**	**SE**	**t-value**	**p**_**holm**_
Π/4 vs Π/2	−1.105	0.481	−2.298	0.028
Π/4 vs 3Π/4	−2.350	0.481	−4.887	<0.001
Π/4 vs Π	−3.743	0.481	−7.785	<0.001
Π/2 vs 3Π/4	−1.245	0.481	−2.590	0.028
Π/2 vs Π	−2.638	0.481	−5.487	<0.001
3Π/4 vs Π	−1.393	0.481	−2.897	0.020
Dartboard vs RMS Dartboard	2.105	0.572	3.579	0.003
Dartboard vs Windmill	0.990	0.572	1.729	0.098
RMS Dartboard vs Windmill	3.095	0.572	5.408	<0.001

**Post Hoc Test**				
**N135**	**Mean Δ**	**SE**	**t-value**	**p**_**holm**_
Π/4 vs Π/2	0.255	0.337	0.758	1.000
Π/4 vs 3Π/4	−0.323	0.337	−0.960	1.000
Π/4 vs Π	0.094	0.337	0.280	1.000
Π/2 vs 3Π/4	−0.578	0.337	−1.717	0.572
Π/2 vs Π	−0.161	0.337	−0.478	1.000
3Π/4 vs Π	0.417	0.337	1.240	1.000
Dartboard vs RMS Dartboard	1.440	0.632	2.280	0.033
Dartboard vs Windmill	2.465	0.632	3.903	0.002
RMS Dartboard vs Windmill	3.905	0.632	6.183	<0.001

**Post Hoc Test**				
**P240**	**Mean Δ**	**SE**	**t-value**	**p**_**holm**_
Π/4 vs Π/2	−0.374	0.191	−1.956	0.192
Π/4 vs 3Π/4	0.199	0.191	1.042	0.610
Π/4 vs Π	0.393	0.191	2.055	0.192
Π/2 vs 3Π/4	0.573	0.191	2.998	0.026
Π/2 vs Π	0.767	0.191	4.011	0.002
3Π/4 vs Π	0.194	0.191	1.013	0.610
Dartboard vs RMS Dartboard	−0.444	0.298	−1.448	0.151
Dartboard vs Windmill	−1.359	0.298	−4.556	<0.001
RMS Dartboard vs Windmill	−1.802	0.298	−6.044	<0.001

N75 amplitude did not differ between the three patterns nor between phase angles. P100 amplitude did not differ between the windmill and regular dartboard but differed between the two dartboards and between windmill and RMS dartboard. Differences in P100 amplitude were present between phase angle Π/4 & 3Π/4, Π/4 & Π and between Π/2 & Π but not between the remaining phase angles. N135 amplitude to the two dartboards exceeded that to the windmill but did not differ between the two dartboards. Its amplitude did not differ across phase angles. P240 amplitude differed between the windmill and the two dartboards but not between the two dartboards. Its amplitude differed between phase angles Π/2 & Π but not between the remaining phase angles.

### 3.3 Comparing VEP component latency

**Fig. 5:**
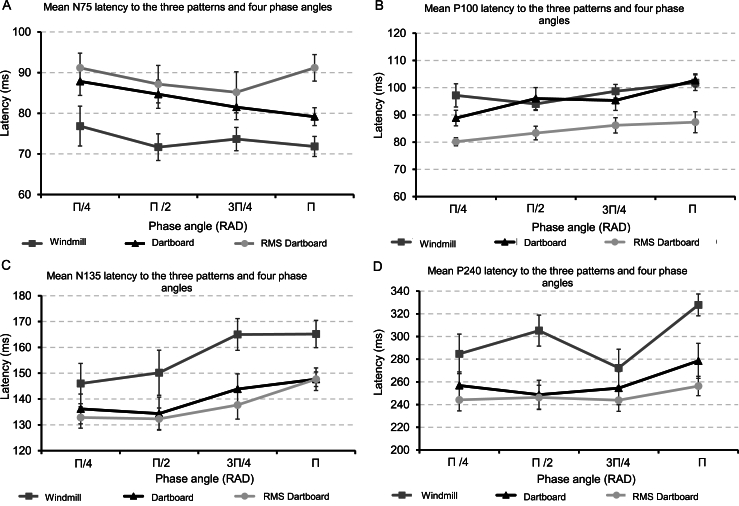
Panel A shows the latencies of the N75 VEP component to the three patterns when rotated through the four angles. Panel B shows the latencies of the P100 VEP component to the three patterns when rotated through the four angles. Panel C shows the latencies of the N135 VEP component to the three patterns when rotated through the four angles. Panel D shows the latencies of the P240 VEP component to the three patterns when rotated through the four angles. The error bars indicate the standard error of the mean.

Panels A–D of Fig. 5 show the grand mean latency of the four VEP components to the three patterns at each phase angle.

Table 2 contains the results of the ANOVA for repeated measures of individual latencies of the four VEP components to each of the three patterns.

**Table 2:** The table holds the results from the repeated measures ANOVA of individual VEP component latency to the three pattern and four phase angles. Violation of sphericity was corrected by adjusting the degrees of freedom using the method of Huynh-Feldt. Partial ETA squared (η_p_^2^) served as a measure of effect size.

	F	df	df Residuals	p	η_p_^2^
**N75**					
PATTERN	15.927	1.884	20.728	<0.001	0.591
PHASE ANGLE	1.763	2.937	32.309	0.028	0.138
PATTERN*PHASE ANGLE	0.701	3.508	38.587	0.021	0.060
**P100**					
PATTERN	14.999	2.000	23.744	<0.001	0.577
PHASE ANGLE	8.760	3.000	36.236	<0.001	0.433
PATTERN*PHASE ANGLE	1.323	5.246	57.708	0.266	0.107
**N135**					
PATTERN	12.424	1.676	18.434	<0.001	0.530
PHASE ANGLE	7.473	2.912	32.030	<0.001	0.405
PATTERN*PHASE ANGLE	0.487	3.200	35.201	0.705	0.042
**P240**					
PATTERN	13.509	1.164	12.799	0.002	0.551
PHASE ANGLE	4.126	3.000	37.284	0.014	0.273
PATTERN*PHASE ANGLE	1.089	5.441	59.855	0.378	0.090

N75 latency remained the same across phase angle but differed between the three patterns. No two-way interaction between pattern and phase angle was present. There was a change in P100 latency between the three pattern and across phase angle. No two-way interaction between the three pattern and phase angle was present. There was a change in N135 and P240 latency between the pattern, but only N135 latency changed across phase angle. No two-way interaction between the factors PATTERN and PHASE ANGLE was present.

Table 2a contains the results of our post-hoc comparison of component latency to the different conditions.

**Table 2a:** Result of post hoc analysis of VEP component latency at the different phase angles and patterns. All p-values adjusted for comparing a family of 6 phase angles or 3 patterns using the method Holm-Bonferroni. Phase angle results are averaged over PATTERN and pattern results are averaged over PHASE ANGLE.

Post Hoc Test				
N75	Mean Δ	SE	t-value	p_holm_
Π/4 vs Π/2	4.111	2.498	1.646	0.437
Π/4 vs 3Π/4	5.167	2.498	2.068	0.279
Π/4 vs Π	4.566	2.498	1.823	0.386
Π/2 vs 3Π/4	1.056	2.498	0.423	1.000
Π/2 vs Π	0.444	2.498	0.178	1.000
3Π/4 vs Π	−0.611	2.498	−0.245	1.000
Dartboard vs RMS Dartboard	−5.375	2.725	−1.973	0.061
Dartboard vs Windmill	−9.792	2.725	−3.593	0.003
RMS Dartboard vs Windmill	−15.167	2.725	−5.566	<0.001

**Post Hoc Test**				
**P100**	**Mean Δ**	**SE**	**t-value**	**p**_**holm**_
Π/4 vs Π/2	−2.389	1.739	−1.373	0.358
Π/4 vs 3Π/4	−4.667	1.739	−2.683	0.045
Π/4 vs Π	−8.556	1.739	−4.919	<0.001
Π/2 vs 3Π/4	−2.278	1.739	−1.310	0.358
Π/2 vs Π	−6.167	1.739	−3.545	0.006
3Π/4 vs Π	−3.889	1.739	−2.236	0.097
Dartboard vs RMS Dartboard	11.458	2.769	4.277	<0.001
Dartboard vs Windmill	2.208	2.769	0.824	0.419
RMS Dartboard vs Windmill	13.667	2.769	5.101	<0.001

**Post Hoc Test**				
**N135**	**Mean Δ**	**SE**	**t-value**	**p**_**holm**_
Π/4 vs Π/2	−0.611	3.872	−0.158	0.876
Π/4 vs 3Π/4	−10.500	3.872	−2.712	0.042
Π/4 vs Π	−15.167	3.872	−3.917	0.003
Π/2 vs 3Π/4	−9.889	3.872	−2.554	0.046
Π/2 vs Π	−14.556	3.872	−3.760	0.003
3Π/4 vs Π	−4.667	3.872	−1.205	0.473
Dartboard vs RMS Dartboard	2.875	4.099	0.701	0.490
Dartboard vs Windmill	16.083	4.099	3.923	0.001
RMS Dartboard vs Windmill	18.958	4.009	4.625	<0.001

**Post Hoc Test**				
**P240**	**Mean Δ**	**SE**	**t-value**	**p**_**holm**_
Π/4 vs Π/2	−4.778	9.375	−0.510	1.000
Π/4 vs 3Π/4	5.056	9.375	0.539	1.000
Π/4 vs Π	−25.611	9.375	−2.732	0.050
Π/2 vs 3Π/4	9.833	9.375	1.049	0.905
Π/2 vs Π	−20.833	9.375	−2.222	0.133
3Π/4 vs Π	−30.667	9.375	−3.271	0.015
Dartboard vs RMS Dartboard	12.000	10.008	1.199	0.243
Dartboard vs Windmill	37.833	10.008	3.780	0.002
RMS Dartboard vs Windmill	49.833	10.008	4.980	<0.001

N75 latency was shorter for the windmill than the two dartboards. P100 latency changed between the phase angle Π & Π/2 as well as Π & Π/4. Its latency to the RMS dartboard was shorter compared to the windmill and regular dartboard but P100 latency did not change between the latter two. N135 latency was longer to the windmill than to the two dartboards. Its latency did not change between phase angle Π/4 & Π/2 and 3Π/4 & Π. Its latency changed between phase angles 3Π/4 & Π/2, 3Π/4 & Π/4, Π & Π/4 & Π & Π/2. P240 latency was longer to the windmill than to the two dartboards but did not change between the latter. It changed between phase angle 3Π/4 & Π and 3Π/4 & Π/4 but not between any of the remaining phase angles.

### 3.4 Source localisation

**Fig. 6:**
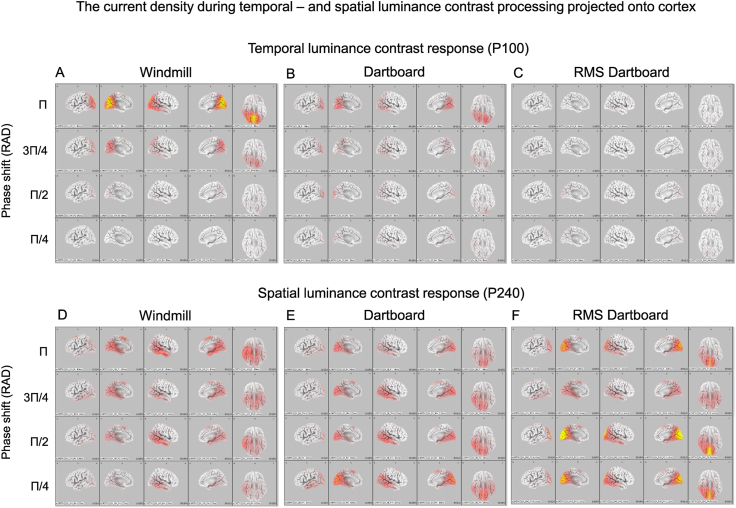
Panels A–C show the distribution of the current source density during the P100 VEP component to the three patters at the four phase angles. This component reflected the neuronal activity during the neuronal luminance component. Panels D–F show the distribution of the current source density during the P240 VEP component to the three patterns at the four phase angles. This component reflected the neuronal activity during the neuronal contrast component.

Panels A–D of Fig. 6 show the current density across cortex during temporal luminance contrast processing. It peak was located at the occipital pole. Panels D–F of Fig. 6 show the current density across cortex during spatial luminance contrast processing. Its peak was located in calcarine sulcus but extended to cuneus, fusiform and inferior temporal cortex.

**Fig. 7:**
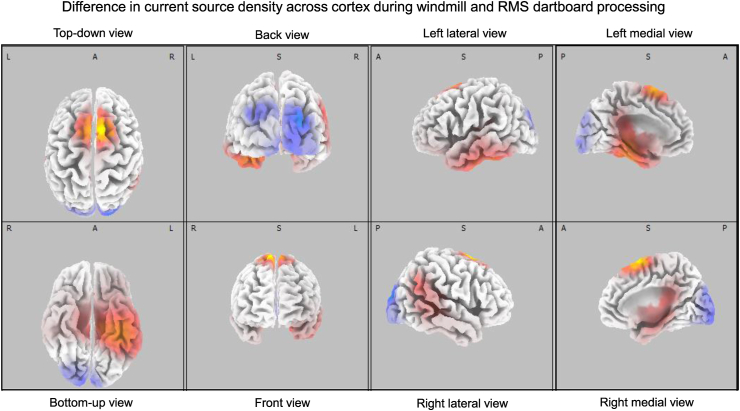
The figure shows the significant differences in CSD rendered onto a standardised cortical surface. The areas coloured yellow, orange and red indicate cortex where CSD during processing of the windmill pattern exceeded that during processing of the RMS-dartboard. The areas coloured in shades of blue indicate cortex where CSD during processing of the RMS-dartboard exceeded that during processing of the windmill.

Fig. 7 shows the result of the statistical comparison of the current density across cortex during processing of the windmill and the RMS dartboard. Cortical regions where CSD during processing of the windmill was higher are shown in shades of blue. Regions where CSD was higher during processing of the RMS dartboard are shown in yellow, orange and red. During processing of the windmill CSD was higher in lingual gyrus, cuneus and middle occipital cortex than during processing of the RMS dartboard. During processing of the RMS dartboard CSD was higher in fusiformis, inferior temporal cortex and supplemental motor area.

### 3.5 Time-frequency analysis

**Fig. 8:**
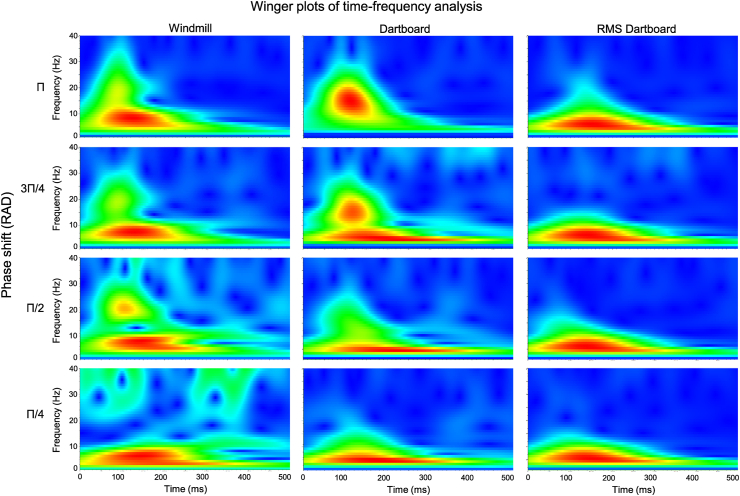
The six panels depict the time-frequency composition of the oscillations in the electric potential to each stimulus in the form of Winger plots. The left column depicts those obtained to the windmill pattern at the four phase angles. The middle column depicts those obtained to the dartboard pattern at the four phase angles. The right column depicts those obtained to the RMS dartboard pattern at the four rotations angle. The presence of high temporal frequency oscillations (β-band) during processing of the windmill and dartboard pattern by the neuronal luminance component are apparent at the larger phase angle. Low temporal frequency oscillations (δ-band) during processing of the two dartboards pattern by the neuronal contrast component are apparent at all phase angle.

Fig. 8 depicts the time-frequency composition of the VEP to the three patterns at the four phase angles in the form of Winger plots. Faster oscillations in the VEP were prominent during the initial neuronal response reflecting the relative size of the stimulus experiencing an increase in luminance; consistent with a response based on a mechanism selective to temporal luminance contrast. Oscillations in this frequency band increased in prominence as phase angle increased but subsided before the next image exchange. Slower oscillations in the VEP were prominent during the neuronal response reflecting the number of contrasts per unit area; consistent with a response based on a mechanism selective to spatial luminance contrast. These slower oscillations were unaffected by phase angle and persisted until the next image exchange.

Table 3 contains the results of our statistical comparison of the power in the alpha and beta band using a repeated measures ANOVA. Power in the alpha band did not differ between patterns nor across phase angle. A to-way interaction between the factors PATTERN and PHASE ANGLE was present. Power in the beta band did not differ between patterns but increased with increasing phase angle. A two-way interaction between the factors PATTERN and PHASE ANGLE was present.

**Table 3:** The table holds the results from the repeated measures ANOVA of power in alpha and beta band to the three pattern and four phase angles. Violation of sphericity was corrected by adjusting the degrees of freedom using the method of Huynh-Feldt. Partial ETA squared (η_p_^2^) served as a measure of effect size.

	F	df	df Residuals	p	η_p_^2^
**Alpha band**					
PATTERN	2.949	1.951	21.462	0.075	0.211
PHASE ANGLE	5.455	1.397	15.368	0.024	0.332
PATTERN*PHASE ANGLE	4.520	1.773	19.507	0.005	0.291
**Beta band**					
PATTERN	8.211	1.174	12.911	0.011	0.427
PHASE ANGLE	10.359	1.170	12.867	0.005	0.485
PATTERN*PHASE ANGLE	5.863	2.199	24.186	0.007	0.348

## 4 Discussion

### 4.1 Summary of main findings

N75 amplitude remained the same level across the three patterns and four phase angles. P100 amplitude to the windmill and regular dartboard increased with phase angle. We therefore reject H0 in favour of H1 of our Hypothesis 1. P100 amplitude to the windmill exceeded that to the regular dartboard, particularly at larger phase angles. We therefore reject H0 in favour of H1 of our Hypothesis 2. P100 amplitude to the windmill and regular dartboard pattern exceeded that to the RMS dartboard. A two-way interaction between phase angle and pattern was observed for P100 amplitude. N135 amplitude from the RMS and regular dartboard exceeded that from the windmill but was invariant across phase angle. P240 amplitude from the two dartboards exceeded that from the windmill. Its amplitude decreased with increasing phase angle for the windmill and regular dartboard. P240 amplitude also dropped with increasing phase angle; a response not observed in N135. N75 latency did not change across patterns or phase angle. P100, N135 and P240 latency increased with phase angle in all three patterns. P100 latency from the RMS dartboard was shorter than from the dartboard or windmill, but in the latter two it increased with increasing phase angle. N135 and P240 latencies from the two dartboards were shorter than from the windmill and increased with increasing phase angle.

Our source localisation revealed that during temporal luminance contrast processing, the highest current density was located in Cuneus and Precuneus. This points to areas of the dorsal processing stream as the origin of re-entrant projections modulation of the neuronal response in V1. During spatial luminance contrast processing the highest current density was located in inferior temporal cortex. This points to areas of the ventral processing stream as the origin of re-entrant projections modulating the neuronal response in V1. Oscillations in the beta band were prominent during temporal luminance contrast processing. Power in this band did not vary between patterns but increased with phase angle. Oscillations in the alpha band VEP were prominent during spatial luminance contrast processing. Power in this band did not vary between patterns and across phase angles.

### 4.2 Relative contribution of V1 and area V2 to the neuronal response captured by the VEP

By limiting our analysis to the amplitudes and latencies of components of the VEP recorded at electrode Oz, we captured the waxing and waning of the neuronal response of the same patch of V1 [12]. Located at the pole of occipital cortex, this electrode covers a patch of visual cortex that receives its input from the fovea [57]. The vertical meridian divides the fovea. Its cortical representation runs the V1–V2 border [58]. There is a large inter-individual variation in the location of this border. This section will show that the two areas are anatomically and functionally sufficiently similar as to render variation in their relative contribution to the VEP immaterial. For the two areas to influence the VEP their common border must be located within the cortex underlying electrode Oz. An imbalance in size of active neuronal population is improbable as an explanation, as the surface area of area V2 is between 75 and 90 % of the surface area of V1 [59]. Hence, the number of neurons active during retinal input processing will be similar. In both areas neurons selectively responding to motion, orientation or chromatic content congregate in sub-regions with distinct affinity for cytochrome oxidase specific staining; “blobs” in V1 [60], “stripes” in area V2 [61]. Lastly, neuronal activity in V1 is modulated by re-entrant projections originating in area V2 within 14 ms [26]. Given the extensive interconnections between the two areas, their respective neuronal response will rapidly converge. The two areas are anatomically and functionally sufficiently similar and the activity converges sufficiently fast, so that their relative contribution to the VEP can be considered identical.

### 4.3 Relative contribution of magno-, parvo- and koniocellular input to the neuronal response in V1 captured by the VEP

Primary visual cortex (V1) receives three types of retinal input carried by magno-, parvo- and koniocellular neurons. Koniocellular neurons respond to chromatic contrast only [62], so that their contribution to the neuronal response to our pattern defined by luminance contrast only will be negligible. Hence, this section will focus on the contributions of magno- and parvocellular neurons to the neuronal response recorded by the VEP. Our earlier work using dartboard and windmill pattern noted that N75 and P100 amplitude increased with relative stimulus area undergoing a reversal in luminance. Given the retinotopic organisation of V1, we concluded that they reflect the size of the neuronal population activated by the change in luminance. N135 and P240 amplitude reflected the number of contrast edges per unit area [48] and with it the size of the active neuronal population processing these [31,43,46]. In a study increasing the luminance gradient of the edges in a series dartboard patterns, N75 and P100 amplitude increased in a non-linear manner with luminance gradient, tailoring off when the luminance gradient was above the threshold of magnocellular neurons [63]. N135 and P240 amplitude increased linearly with luminance gradient [45]. A non-linear response, saturating at low luminance is a characteristic of magnocellular neurons, while a linear response is a characteristic of parvocellular neurons.

Receptive field size of retinal ganglion cells increases with eccentricity, paralleling a decline in visual acuity [[64], [65], [66], [67], [68], [69]]. The retinal input from the fovea occupies between 3 and 6 times as much area in human V1 than that originating outside of the fovea [70], an over-representation termed ‘cortical magnification’. Input from the fovea contains 35 times more parvocellular than the magnocellular axons, a ratio that drops to 5:1 at 15° eccentricity [71]. Compensated for receptive field size, parvo- and magnocellular neurons are equally adept at resolving spatial luminance contrast [72,73]. Consequently, the neuronal response captured in the VEP at electrode Oz is driven by activity from within the fovea and hence to a greater extent by parvo-rather than magnocellular neurons [74].

Processing the retinal input involves iterative interactions between striate and extra-striate areas [27]. In such an interconnected system, the neuronal response in striate cortex is as much the result of an interaction between striate cortex and individual extra-striate areas as well as the interaction between different extra-striate areas. Re-entrant connection with fast conducting axons will exert their influence on the ongoing neuronal response more rapidly. A fast change in neuronal response results in a faster change in the electric potential at the scalp which manifests as high temporal frequencies in the VEP. The presence of high temporal frequencies in the neuronal response captured by N75 and P100 is consistent with this response being modulated by re-entrant projections with axons with fast conduction velocities. The presence of slow temporal frequencies in the neuronal response captured by N135 and P240 is consistent with the response being modulated by axons with slow conduction velocities. This concurs with the observation that high temporal frequencies in the VEP are more prominent during processing of low spatial frequency and vice versa [56]. Axonal conduction velocities of magnocellular neurons are faster than that those of parvocellular neurons, so that the neuronal response during retinal input processing is initially modulated by magnocellular - followed by parvocellular neurons. The temporal frequency composition of the VEP is consistent with the neuronal response during temporal luminance contrast processing being modulated by re-entrant projections with fast axonal conduction velocities, the neuronal response during spatial luminance contrast processing by re-entrant projections with slower axonal conduction velocities. Fast axonal conduction velocity is a characteristic of magnocellular neurons, while slower axonal conduction velocities is a characteristic of parvocellular neurons.

Sampling temporal luminance contrast at any point across the visual field requires a single detector. Sampling spatial luminance contrast however requires numerous detectors; i.e. to sample 360° with a resolution of 10° involves 36 detectors. This is exemplified by orientation selectivity in V1 of many species being organised in a pinwheel fashion [75]. The difference in the number of magno-compared to parvocellular neurons is consistent with their respective role in processing temporal – and spatial luminance contrast.

Linking magnocellular neurons to temporal - and parvocellular neurons to spatial luminance contrast processing leads to a paradox between their response characteristics and the theoretical properties of a temporal – and spatial luminance contrast detector. For a temporal luminance contrast detector to signal a wide range of luminance change, its responds level should vary linearly with luminance, i.e. exhibit a linear response characteristic. However, a linear response to luminance it is a characteristic of parvocellular neurons. Conversely, for a detector to reliably detect a spatial luminance contrast its response level should saturate at low luminance contrast, i.e. exhibit a non-linear response to luminance. A non-linear response to luminance is a characteristic of magnocellular neurons. There appears to be a transposition between stimulus selectivity and response characteristic of magno- and parvocellular neurons and the neuronal response captured by the VEP during temporal and spatial luminance contrast processing.

### 4.4 The origin of re-entrant projections and their influence on the neuronal response

Our temporal frequency analysis of the VEP revealed that the neuronal response in V1 is modulated by re-entrant projections with fast axonal conduction during temporal luminance contrast processing followed by re-entrant projections with slow axonal conduction velocities during spatial luminance contrast processing. Successive activation of cortical areas occurs faster along the dorsal processing stream than along the ventral processing stream [76]. This points to the former being interconnected by neurons with fast conducting axons and the latter by neurons with slow conducting axons. During temporal luminance contrast processing our source localisation placed the highest CSD in areas of the dorsal stream and during spatial luminance contrast processing in areas of the ventral stream. This section examines the presence of other characteristics of the VEP components are consistent with properties of the neuronal response in the two processing streams. The retinotopic organisation of V1 translates relative stimulus area changing from black to white into neuronal population size activated by this increase in luminance. For P100 amplitude to signal the relative stimulus area increasing in luminance, re-entrant projections must conserve the spatial distribution of neurons in V1 activated by the thalamic input. Re-entrant projections between cortical areas with a retinotopic organisation are less diffuse than those between cortical areas lacking such an organisation [77]. Areas of the dorsal processing stream sport a retinotopic representation of at least part of the contralateral visual field [78,79], while areas of the ventral processing stream exchange a retinotopic organisation in favour of an organisation based on features and object identity [[80], [81], [82], [83]]. In contrast. N135 and P240 amplitude reflect the pattern, i.e. windmill or dartboard, rather than stimulus area changing from black to white. We interpret this to indicate that re-entrant projections are no longer constraint by retinotopy and carry information on object identity. There are parallels between the neuronal response during temporal- and spatial luminance contrast processing, properties of magno- and parvocellular neurons and characteristics of cortical areas of the dorsal - and ventral processing stream. These observations concur with our source localisation that during spatial luminance contrast processing the site of highest current density is located in areas of the dorsal processing stream and during spatial luminance contrast processing in areas of the ventral processing stream (See Fig. 7) [84].

### 4.5 Limitations, loose ends and open issues

In this final section, we will highlight some issues influencing the wider relevance of our findings and some issues that merit addressing in future studies.

The first issue concerns the general relevance of our findings as it is based on measurements of a dozen participants. While this may limit the ability to generalise our findings, the findings reported are consistent with our previous work. The second, concerns the accuracy and reliability of our source localisation, given that is was based on 32 electrodes. KEY-LORETA is provides an accuracy of 10 mm in localising the source of an electric signal using 16 electrodes [13] Doubling the number of electrodes does increase accuracy or reliability of the source localisation using KEY-LORETA, however it does so with diminishing returns, i.e. doubling the number of electrodes does not double accuracy or reliability. The use of 32 electrodes leaves our source localisation with sufficiently accurate to identify the Brodmann area of a signal [85]. Our observation that the neuronal origin of our signals were restricted to occipital cortex is in agreement with work involving subdural recording during surgery in man [86] and monkey [87] reporting little activity to pattern reversing stimuli beyond area V2. The third, is the difference in finding between the present study and our previous investigations. In previous investigations we observed that for the same population size active during temporal luminance contrast processing P100 amplitude was smaller if the population size processing spatial luminance contrast was larger. Similarly, for the same neuronal population size active during spatial luminance contrast processing N135 amplitude was smaller when the size of the neuronal population active during temporal luminance contrast processing was larger [31,[43], [44], [45], [46]]. In our present study, its amplitude did not differ between these patterns. Also, for the same neuronal population size active during temporal luminance contrast processing, P100 latency was shorter when the neuronal population active during spatial luminance contrast processing was larger. Conversely, for the same neuronal population size active during spatial luminance contrast processing, N135 latency was longer when the neuronal population active during temporal luminance contrast processing was larger. No such difference in P100 and N135 amplitude and latency was observed in the present study. Whether this difference is accounted for by the relative small number of participants measured is uncertain but in our opinion unlikely. It is interesting to note that the influence of dipole interaction on VEP components that we reported in our previous work involved either variation of a dartboard pattern [31,44,45] or variation of a windmill pattern [43]. Clarifying this and the previous issues merits further investigation. The forth, is the re-emergence of a neuronal response linked to temporal luminance contrast processing during a neuronal response processing spatial luminance contrast. This response resulted in P240 amplitude decreasing linearly with increasing phase angle. N135 amplitude showed no such reaction, indicating that this neuronal response emerged later. The presence of this neuronal response is unexpected as our previous work concluded that the neuronal response associated with temporal luminance contrast processing is phasic in nature [31,[43], [44], [45], [46]]. Reactivation of neuronal responses in V1 has been reported in the monkey [33] and humans [32]. These studies however linked the reactivated neuronal response following object closure, while the characteristic of the reactivated neuronal response in our study preceded object closure. The reactivated neuronal response rendered P240 less positive. This suggests that it either arises due to activation of supragranular laminae by re-entrant projections or is inhibitory in nature. This is also an issue that merits future investigation. A final issue is the presence of neuronal response in supplementary motor area (SMA). The presence of this neuronal response is unexpected as the only motor response required by our paradigm was fixation. The role of this neuronal response in relation to our paradigm is unclear, as no motor response was require from our participants.

## 5 Conclusions

From the findings of our present investigation, we conclude that both amplitude and latency of VEP components arise from independent neuronal responses processing temporal – and spatial luminance contrast. It also corroborates findings form or previous work that the neuronal response during temporal luminance contrast processing is subject to modulation by re-entrant projections with fast axonal conduction velocities originating in areas of the dorsal processing stream. And that the neuronal response during spatial luminance contrast processing is subject to modulation by re-entrant projections with slow axonal conduction velocities originating in areas of the ventral processing stream. We failed to observe any influence linked to dipole interaction on the VEP as noted in our previous work. The role played by pattern in the manifestation of dipole interaction on the VEP is something that needs to be addressed in future work. Having set out to investigate the influence of spatial luminance contrast signal on early visual processing, i.e. P100, we discovered an influence of temporal luminance contrast signal on late visual processing, i.e. P240.

## Financial disclosure

The work reported in this article was performed as an investigator-initiated trial (IIT) without financial support from a third party.

## Data availability statement

We are willing to provide the dataset through a Data Transfer Agreement to any legitimate researcher seeking access for replication analysis. Requests for data sharing or access should be formally submitted to the Data Governance Board of the University Hospital Zürich at data-governance@usz.ch or the Research Data Service Centre of the Clinical Trials Centre at the University Hospital Zürich via email at ctc-rdsc@usz.ch.

## CRediT authorship contribution statement

**Valentine L. Marcar:** Writing – review & editing, Writing – original draft, Validation, Software, Project administration, Methodology, Investigation, Formal analysis, Conceptualization. **Martin Wolf:** Writing – review & editing, Validation, Resources.

## Declaration of competing interest

The authors declare the following financial interests/personal relationships which may be considered as potential competing interests:Valentine L. Marcar has patent #International Patent Number: PCT/EP2022/061870 issued to None. If there are other authors, they declare that they have no known competing financial interests or personal relationships that could have appeared to influence the work reported in this paper.

## List of abbreviations

ANOVA: Analysis of Variance
BA: Broadman Area
CSD: Current Source Density
DB: Dartboard
DC: Direct Current
EEG: Electroencephalography
ERP: Event Related Potential
HDD: Hard Disk Drive
Hz: Hertz
ICA: Independent Component Analysis
ISCEV: International Society for Clinical Electrophysiology of Vision
PC: Personal Computer
sLORETA-KEY: Standardized Low Resolution Brain Electric Tomography- Key Institute Zürich
RMS: Root-Mean Squared
SMA: Supplementary Motor Area
V1: Primary Visual Cortex
V2: Secondary Visual Cortex
VEP: Visual Evoked Potential
WM: Windmill
